# Media representation of tobacco control in China: A comparative analysis of agenda-setting across different policy contexts during 2017–2022

**DOI:** 10.18332/tid/204741

**Published:** 2025-07-03

**Authors:** Yu Chen, Yujiang Cai, Xinrui Yang, Xinyao Yu, Rui Zhang, Heng Zhang, Jing Xu, Kin-Sun Chan

**Affiliations:** 1 School of Art and Communication, Fujian Polytechnic Normal University, Fuqing, China; 2 School of International Studies, Peking University, Beijing, China; 3 Faculty of Social Sciences, University of Macau, Macau, China; 4 Faculty of Humanities and Arts, Macau University of Science and Technology, Macau, China; 5 School of Journalism and Communication, Peking University, Beijing, China

**Keywords:** news coverage, tobacco control policy, agenda-setting theory, co-word analysis, topic model

## Abstract

**INTRODUCTION:**

While media play a crucial role in tobacco control policy advancement, little is known about how different policy contexts shape media coverage of tobacco control, particularly in China’s unique political and administrative systems. This study innovatively compares tobacco control news coverage across three regions with distinct tobacco control policy environments: mainland China, Guangdong Province, and Macao SAR, examining how policy contexts influence media agenda-setting before and during the COVID-19 pandemic.

**METHODS:**

Using the WisersOne database, we analyzed 749 tobacco control news articles from three influential newspapers (*People’s Daily, Nanfang Daily, and Macao Daily News*) from 2017–2022. We employed a mixed-methods approach combining co-word analysis and topic modeling using Python. The analysis was divided into pre-pandemic (2017–2019) and pandemic (2020–2022) periods, to examine temporal changes in media coverage patterns and policy priorities.

**RESULTS:**

Significant disparities in coverage intensity and thematic focus were found across regions. *Macao Daily News* published substantially more tobacco control articles (596) than *People’s Daily* (46) and *Nanfang Daily* (107). While mainland media primarily focused on youth tobacco prevention and World No Tobacco Day, Macao’s coverage demonstrated more comprehensive themes including enforcement, legislative participation, and addiction prevention. During the pandemic, all regions showed reduced coverage but maintained distinct thematic priorities, with emerging emphasis on e-cigarette regulation and youth protection.

**CONCLUSIONS:**

This study reveals how policy environments substantially influence media agenda-setting in tobacco control. Macao’s comprehensive tobacco control legislation corresponds to more frequent and diverse media coverage, while mainland China’s limited national smoke-free legislation is reflected in sparse, fragmented coverage. These findings suggest the need to strengthen media advocacy strategies in mainland China to advance national tobacco control policies, particularly by leveraging successful examples from regions with strong tobacco control measures.

## INTRODUCTION

### Different tobacco control policies in China’s regions

The tobacco epidemic represents a significant public health challenge globally. In response, the World Health Organization (WHO) introduced the Framework Convention on Tobacco Control (FCTC) and MPOWER measures to assist countries in implementing effective tobacco control strategies^1-4^. In 2005, China endorsed the WHO FCTC, marking a significant development in its tobacco control policies. Despite some progress, China’s tobacco control legislation remains less advanced compared to international standards. As of 2019, WHO evaluated China’s smoke-free regulations at Level IV, indicating the lowest compliance^2^.

Macau SAR, recognized as a healthy city by WHO, has progressively enhanced its legal framework for tobacco control through revisions of the ‘Regime of Tobacco Prevention and Control’ in 2012, 2018, and 2022^3^. The implementation has been effective, as evidenced by the decreasing trend in tobacco usage from 16.9% in 2011 to 10.6% in 2022^4^.

### Research on Chinese news media reporting

Research on tobacco control media coverage in China primarily employs content and text analysis methods. Previous studies have analyzed media discourse, opinion leaders, and tobacco control culture^5,6^. Zheng et al.^7^ evaluated newspaper articles using the WHO MPOWER framework. Studies have also examined the relationship between media and policy agendas in tobacco control^8,9^.

### Agenda-setting theory

Agenda-setting theory, introduced by McCombs^10^, posits that mass media play a crucial role in setting the public agenda^10-14^. The theory explores interactions between media, public, and policy agendas through a three-part linear process. Media significantly affect the content and value orientation of public opinion on various issues. Research has established a positive correlation between media agenda and public policy agenda^15^.

This study examines the variance in tobacco control reporting within mainstream newspapers across different regulatory environments in various regions of China, through the lens of agenda-setting theory. It offers evidence and recommendations for enhancing tobacco control advocacy based on this theory.

## METHODS

### Data sources and collection

Based on previous research approaches, this study utilized WisersOne (http://www.wisers.com), the largest and most authoritative database of Chinese newspapers, to source pertinent news articles. Specifically, articles were retrieved using ‘tobacco control’ (控烟) as the search keyword from three strategically selected newspapers: *People’s Daily*, *Nanfang Daily*, and *Macao Daily News*. We included all news articles, editorials, opinion pieces, and feature stories that substantially addressed tobacco control topics. Two researchers independently reviewed all articles to ensure they substantially addressed tobacco control policies, implementation, or related public health issues. Any disagreements were resolved through discussion with a third researcher.

The selection of these three newspapers was based on their distinct administrative levels and significant influence (Table 1). The *People’s Daily*, as the organ of the Central Committee of the Communist Party of China, boasts a daily circulation of 3.46 million copies^16^. The *Nanfang Daily*, representing the Guangdong Provincial Committee of the Communist Party of China, daily circulates 850000 copies^17^. The *Macao Daily News* holds the status of the most widely circulated and representative daily in Macao SAR^18^. These newspapers serve as official chronicles and previous research demonstrates significant correlation between newspaper reporting and coverage in other media platforms.

**Table 1:** Overview of the three newspapers

*Newspaper*	*Governing Body*	*Influence Scope*	*Circulation*
**People’s Daily**	Central Committee of the Communist Party of China	National	3460000
**Nanfang Daily**	Guangdong Provincial Committee of the Communist Party of China	Provincial	850000
**Macao Daily News**	Government Information Bureau of the Macao Special Administrative Region	Regional	80000

The study period was strategically divided into two phases:

- Pre-pandemic: 1 January 2017 – 31 December 2019
- During the pandemic: 1 January 2020 – 31 December 2022

This temporal division was based on: 1) the COVID-19 pandemic’s significant impact on media priorities, and 2) the implementation of key tobacco control policies during these periods. In 2019, the national Healthy China Action and provincial Healthy Guangdong Action were launched^2^. Macao enacted its New Tobacco Control Law in 2018, followed by significant e-cigarette regulations in 2022^5^.

### Data processing

The analytical framework integrated co-word analysis and topic modeling to examine thematic patterns and their evolution. Following established methodological protocols^19-22^, we employed qualitative axial coding to transform textual data into systematic themes. The coding process utilized both deductive and inductive reasoning, with repeated data review and integration of similar codes. Eigenvector centrality (EC) was calculated to measure keywords’ significance within the policy framework, where higher EC values indicate greater centrality and influence in the semantic network.

To complement the co-word analysis and address potential limitations of keyword-based approaches, we implemented topic modeling using Python. The process involved text preprocessing using Peking University’s *pkuseg* toolkit for word segmentation, followed by stop words removal to enhance analysis accuracy. Initial topic identification was conducted through WordCloud visualization, with subsequent topic extraction and clustering using Latent Dirichlet Allocation (LDA) modeling^23^. This integrated analytical framework enabled systematic examination of both manifest and latent content patterns across different policy contexts and time periods, combining quantitative measures with qualitative thematic analysis.

The qualitative and quantitative components of our analysis were integrated through an iterative process. Initially, qualitative axial coding provided a preliminary understanding of thematic patterns, which informed keyword selection for co-word analysis. The semantic networks generated through co-word analysis then revealed underlying relationships between concepts, highlighting potential thematic clusters that might not have been immediately apparent in the qualitative coding. These insights were subsequently used to refine our topic modeling approach, particularly in interpreting the LDA results. Conversely, the topic modeling results helped validate and expand upon the manually coded themes, identifying additional linguistic patterns and thematic associations. This methodological triangulation enhanced the robustness of our findings by allowing each method to inform and validate the others, providing a more comprehensive understanding of media coverage patterns across different policy contexts.

## RESULTS

### Overview of coverage

The analysis of 749 tobacco control news articles revealed significant disparities in coverage intensity and thematic focus across regions. *Macao Daily News* published substantially more articles (596) than *People’s Daily* (46) and *Nanfang Daily* (107). During the pre-pandemic period (2017–2019), a total of 450 articles from *Macao Daily News*, 65 articles from the *Nanfang Daily*, and 33 articles from *People’s Daily* were collected. Coverage decreased during the pandemic period (2020–2022) to 146, 42, and 13 articles, respectively, as shown in Table 2.

**Table 2:** Statistics of news reports based on article count

	*People’s Daily*	*Nanfang Daily*	*Macao Daily News*
**Pre-pandemic**	33	65	450
**During the pandemic**	13	42	146

### Keyword centrality analysis

Analysis of eigenvector centrality (Table 3) revealed distinct patterns in thematic emphasis across regions and time periods. At the national level, *People’s Daily* maintained high centrality for ‘tobacco control’ (EC=0.529 to 0.546), while ‘health’ gained prominence during the pandemic (EC=0.341 to 0.546). *Nanfang Daily* showed a significant shift from tobacco control-specific discourse (EC=0.501 to 0.299) to broader health initiatives (EC=0.251 to 0.553). *Macao Daily News* demonstrated the most balanced thematic distribution, with consistently lower but more diverse EC values (0.381 to 0.134), reflecting comprehensive coverage. The emergence of ‘e-cigarettes’ (EC=0.406) and sustained focus on ‘youth’ (EC=0.252) during the pandemic indicated responsive policy adaptation.

**Table 3:** Keywords and eigenvector centrality (EC)

*People’s Daily*				*Nanfang Daily*				*Macao Daily News*			
Pre-pandemic*		Pandemic period*		*Pre-pandemic*		*Pandemic period*		*Pre-pandemic*		*Pandemic period*	
*Keyword*	*EC*	*Keyword*	*EC*	*Keyword*	*EC*	*Keyword*	*EC*	*Keyword*	*EC*	*Keyword*	*EC*
**Tobacco control**	**0.529**	**Health**	**0.546**	**Tobacco control**	**0.501**	Tobacco control	0.299	**Macau**	**0.381**	**E-cigarettes**	**0.406**
Health	0.341	Tobacco control	0.338	Health	0.251	**Health**	**0.553**	Tobacco control	0.281	Macau	0.316
Smoking	0.190	Work	0.142	Shenzhen	0.341	Shenzhen	0.101	Illegal smoking	0.272	Youth	0.252
China	0.205	Smoking	0.085	Public places	0.267	Action	0.476	Government	0.350	Health bureau	0.202
Beijing	0.238	Supervision	0.046	Smoking	0.215	E-cigarettes	0.106	Entertainment venues	0.290	Smoking	0.175
**Smoking ban**	**0.109**	Action	0.197	E-cigarettes	0.103	Promotion	0.110	E-cigarettes	0.140	Tobacco control	0.190
Public places	0.122	Hygiene	0.112	World No							
Tobacco Day	0.134	Environment	0.143	Smoking room	0.191	Government	0.187				
Tobacco	0.125	Hebei	0.140	Action	0.132	**Civilized**	**0.049**	Health Bureau	0.167	Health	0.150
Implementation	0.188	Citizens	0.168	Regulations	0.134	Positive	0.064	Health	0.137	Quit smoking	0.151
Youth	0.123	**Students**	**0.058**	**Hygiene**	**0.099**	Education	0.110	**Youth**	**0.116**	**Tobacco**	**0.134**

* Stage one: Pre-pandemic 2017–2019. Stage two: During the pandemic 2020–2022.

### Thematic evolution

The pre-pandemic semantic networks (Supplementary file Figures 1.1–1.3) illustrated regional variations in coverage focus. The *People’s Daily* network centered on national guidance and youth prevention, while *Nanfang Daily* emphasized provincial implementation and smart supervision. *Macao Daily News* exhibited more distributed networks encompassing enforcement, legislation, and public health initiatives (Table 4).

**Table 4:** News themes of tobacco control coverage pre-pandemic: A cross-regional comparison

Newspaper	News themes					
**People’s Daily**	National guidance	Health promotion	Smoking ban in public places	Model city	Tobacco warnings	Smoking harm standards
	Tobacco control	Health	Smoking ban	Shenzhen	Tobacco	Plan
	China	Implementation	Public places	Solicit	Youth	Cigarettes
	Beijing	Action	Environment	Smoking control order	Harm	Content
	Regulations	Life	Indoor	Electronic	Packaging	Nicotine
	World No Tobacco Day	All citizens	Passengers	Economy	Warnings	United States
	Prevention and control	Primary and Secondary school students	Effectiveness	Revision	Images	Standards
	Civilized	Elderly	Inspection	Upgrade	Notices	Food
**Nanfang Daily**	Intelligent supervision	Health personnel training	Graded supervision	Public health issues	Community governance	
	Tobacco control	Service	National	World No Tobacco Day	Action	
	Shenzhen	Personnel	Guangdong Province	Tobacco	Environment	
	Public places	Prevention and control	Beijing	Lung cancer	Residential community	
	Electronic eye	Project	Street	Prevention	Family	
	Smoking control	Exchange	Committee	Pollution	Health promotion	
	Monitor	Base	CPPCC*	Activity	Popularization	
	Traffic	Experience	People’s livelihood	Incidence rate	Masses	
**Macao Daily News**	Law enforcement inspection	Health promotion	Administrative legislation			
	Illegal smoking	Tobacco control	Government			
	Entertainment venues	E-cigarettes	Citizens			
	Smoking room	Health	Cooperation			
	New smoking control law	Youth	System			
	Law enforcement	Promotion	Bill			
	Fines	Prevention	Safety			
	Public places	Environment	Implementation			

* Chinese People’s Political Consultative Conference.

During the pandemic (Supplementary file Figures 2.1 and 2.2), network structures evolved significantly. Mainland media networks showed increased health-centric clustering and emerging emphasis on policy promotion. The *Macao Daily News* network maintained comprehensive coverage while adapting to new challenges, particularly e-cigarette regulation and youth protection (Table 5).

**Table 5:** News themes evolution of tobacco control coverage during pandemic: Regional policy responses

Newspaper	News themes					
**Nanfang Daily**	Policy propaganda	Health action	Civilized city	Legal protection	Pollution control	Model unit
	Tobacco control	Health	Civilization	Legislation	Pollution	Shenzhen
	Propaganda	Environment	City	Public Interest Litigation	Treatment	Investigation
	National	Construction	Life	Provincial	Ecology	Exhibition
	Beijing	Knowledge	Society	Inspection	Enterprise	Fangshan
	WeChat	Smoke-free	Public	Documents	Prevention	Enforcement
	Association	Popularization	Regulations	Protection	Greening	Street Districts
	Video	Comprehensive	Citizenry	Safety	Control	Punishment
**Macao Daily News**	Youth smoking harm	Legislative participation	Law enforcement	Addiction prevention		
	E-cigarettes	Government	Inspection	Health Bureau		
	Youth	Society	Prosecution	Citizens		
	Harm	Service	Illegal	Prevention		
	School	Partnerships	Case	Places		
	Tobacco use	Committee	Entertainment	Alcohol		
	Publicity education	Associations	Illegal smoking	Minors		
	Secondhand smoke	Communication	Smoking control enforcement	Sales		

Topic modeling complemented these network patterns, revealing distinct thematic clusters. National coverage evolved from six pre-pandemic clusters (including national guidance and harm standards) to emphasis on policy integration. Provincial coverage shifted from five clusters centered on supervision to enhanced focus on legislative protection. Macao’s coverage maintained three comprehensive clusters throughout, adapting content while preserving structural balance.

## DISCUSSION

This study reveals the significant impact of policy environments on media agenda-setting in tobacco control across different regions of China. The analysis identified three key findings. First, the COVID-19 pandemic substantially reduced tobacco control coverage across all newspapers, highlighting competition between pandemic response and tobacco control within the public health agenda. Second, Macao’s comprehensive tobacco control legislation corresponded to more frequent and diverse media coverage, even during the pandemic. Third, mainland China’s limited national smoke-free legislation was reflected in sparse, fragmented coverage with repetitive themes.

The quantitative and qualitative differences in coverage reflect varying policy priorities and institutional frameworks. Macao’s proactive tobacco control advocacy, aligned with WHO’s FCTC best practices, generated sustained media attention across multiple dimensions: law enforcement, legislative development, and public education. The emergence of ‘legislative participation’ as a prominent theme highlighted effective multi-stakeholder engagement in Macao’s tobacco control efforts^24^. In contrast, mainland coverage showed limited thematic evolution, primarily focusing on youth health and ceremonial events pre-pandemic, with slight shifts toward policy promotion during the pandemic.

While our temporal analysis focused on the pre-pandemic and pandemic periods, we recognize that significant structural shifts in China’s tobacco control governance predated COVID-19 and likely influenced media coverage patterns. The 2018 institutional reorganization that transferred tobacco control responsibilities from the Ministry of Industry and Information Technology (MIIT) to the newly established National Health Commission (NHC) represented a pivotal shift in regulatory authority^1,2^. This transfer potentially altered enforcement priorities by positioning tobacco control more firmly within the public health domain rather than the industrial sector, which may have influenced how national and provincial media framed tobacco control issues. Additionally, the 2019 online sales ban on e-cigarettes, jointly issued by the State Administration for Market Regulation (SAMR) and the State Tobacco Monopoly Administration, abruptly disrupted the growing e-cigarette market^2,5^. This intervention likely contributed to the emerging emphasis on e-cigarette regulation observed in our analysis, particularly in the *Macao Daily News* and *Nanfang Daily* during the pre-pandemic period. The timing of these policy interventions – occurring just before the pandemic – suggests that their influence on media discourse may have been attenuated by the subsequent public health emergency, which diverted media attention from tobacco control issues across all three regions.

These findings align with agenda-setting theory’s emphasis on the relationship between media priorities and policy development^25^. Media coverage not only reflects policy priorities but also shapes public perception and policy discourse^26^. The comprehensive coverage in Macao demonstrates how robust policy frameworks can sustain media attention and public engagement^27^. The disparity in coverage volume – with *Macao Daily News* publishing nearly 30 times more articles than the *People’s Daily* over six years – suggests significant opportunities for strengthening media advocacy in mainland China.

### Implications

This analysis suggests several implications for tobacco control advocacy. First, comprehensive smoke-free legislation appears crucial for sustaining media attention. Second, multi-stakeholder engagement, as demonstrated in Macao, can enhance both policy implementation and media coverage^28^. Finally, maintaining diverse thematic coverage may better serve public education and policy advancement than focusing on isolated events or campaigns^29^.

### Limitations

While this study provides valuable insights into newspaper coverage, it does not address emerging digital media platforms. Future research could explore the role of social media in tobacco control advocacy, particularly regarding youth engagement. Additionally, investigating the bidirectional relationship between media coverage and policy implementation could further illuminate effective advocacy strategies. Lastly, our focus on three newspapers – one from each administrative level – may not capture the full diversity of media coverage within each region. Each selected newspaper has its distinct editorial focus and audience, which may influence its coverage patterns. This limitation is partially mitigated by the fact that the selected newspapers are official Party publications that often set the agenda for other media outlets in their respective regions.

## CONCLUSIONS

This study provides empirical evidence of how different policy environments substantially influence media agenda-setting in tobacco control across three regions of China. Our findings demonstrate that comprehensive tobacco control legislation corresponds to more frequent, diverse, and sustained media coverage, as exemplified by Macao’s proactive tobacco control advocacy and enforcement approach. In contrast, regions with limited national smoke-free legislation, such as mainland China, show sparse, fragmented coverage with repetitive themes, indicating missed opportunities for public education and policy advancement. The COVID-19 pandemic further revealed the vulnerability of tobacco control coverage to competing public health priorities in regions without robust policy frameworks. These findings underscore the need for strengthening media advocacy strategies in mainland China to advance national tobacco control policies by leveraging successful examples from regions with strong tobacco control measures. Future tobacco control initiatives should prioritize developing comprehensive policy frameworks that can generate sustained media attention across multiple dimensions, including enforcement, legislative development, and public education. Such integrated approaches are essential for maintaining tobacco control as a public health priority, even during competing health crises.

## Supplementary Material



## Data Availability

The data supporting this research are available from the authors on reasonable request.
