# Supplementary material for: Media representation of tobacco control in China: A comparative analysis of agenda-setting across different policy contexts during 2017–2022
Source: Tob Induc Dis. 2025 Jul 3;23:10.18332/tid/204741. doi: 10.18332/tid/204741 (PMC12224183; doi:10.18332/tid/204741)
Supplement: Supplementary file 1 [file TID-23-90-s1.pdf]

Appendix: Supplementary Figures

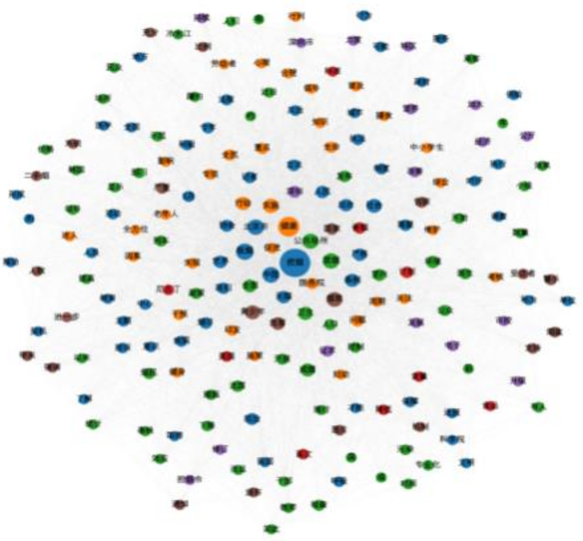

Supplementary Figure 1.1: Semantic Network diagram of Tobacco Control Coverage Pre-pandemic of People's Daily

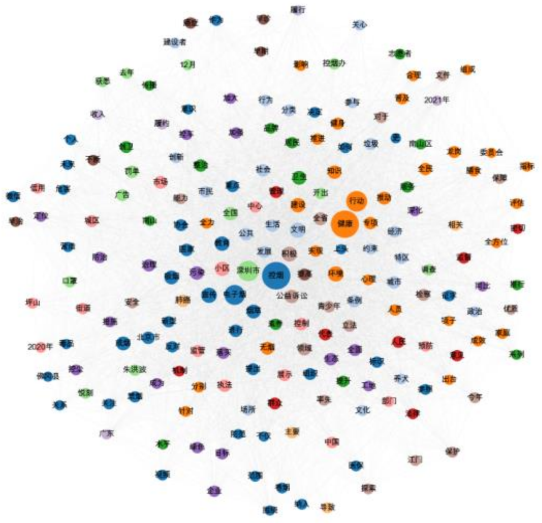

Supplementary Figure 1.2: Semantic Network diagram of Tobacco Control Coverage Pre-pandemic of Nanfang Daily

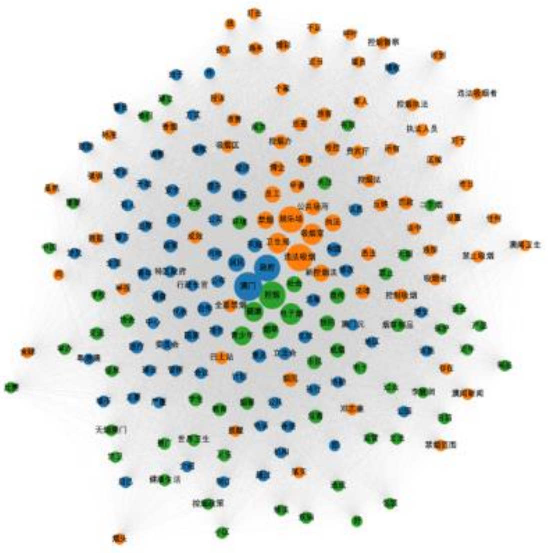

Supplementary Figure 1.3: Semantic Network diagram of Tobacco Control Coverage Pre-pandemic of Macao Daily

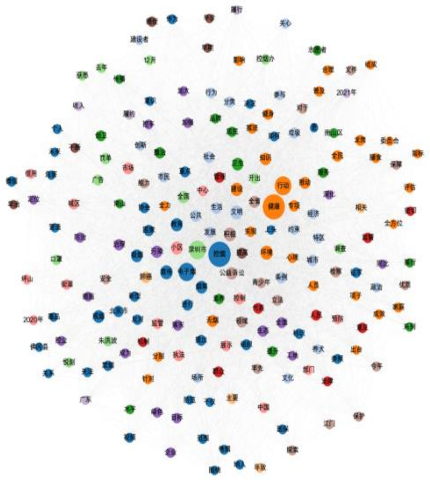

Supplementary Figure 2.1: Semantic Network diagram of Tobacco Control Coverage during pandemic of Nanfang Daily

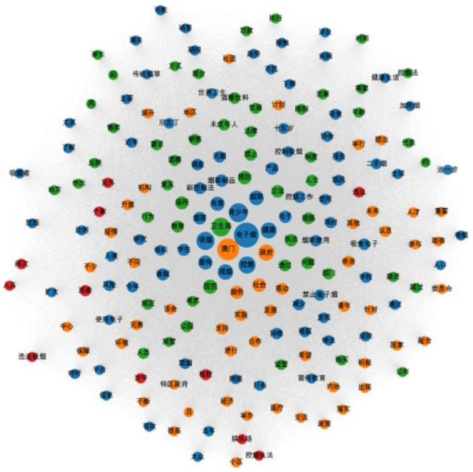

Supplementary Figure 2.2: Semantic Network diagram of Tobacco Control Coverage during pandemic of Macao Daily

© 2025 Chen Y. et al.
